# Biomarkers of Cellular Senescence in Type 2 Diabetes Mellitus

**DOI:** 10.1111/acel.70120

**Published:** 2025-05-30

**Authors:** Caroline Hoong, Joshua N. Farr, David G. Monroe, Thomas A. White, Elizabeth J. Atkinson, Amanda Tweed, Allyson K. Palmer, Nathan K. LeBrasseur, Jad G. Sfeir, Sundeep Khosla

**Affiliations:** ^1^ Division of Endocrinology Mayo Clinic Rochester Minnesota USA; ^2^ Robert and Arlene Kogod Center on Aging Mayo Clinic Rochester Minnesota USA; ^3^ Department of Quantitative Health Sciences Mayo Clinic Rochester Minnesota USA; ^4^ Division of Hospital Internal Medicine Mayo Clinic Rochester Minnesota USA; ^5^ Department of Physical Medicine and Rehabilitation Mayo Clinic Rochester Minnesota USA; ^6^ Section of Geriatric Medicine and Gerontology Mayo Clinic Rochester Minnesota USA

**Keywords:** aging, diabetes, obesity, osteoporosis, senescence

## Abstract

Although animal studies have linked cellular senescence to the pathogenesis and complications of type 2 diabetes mellitus (T2DM), there is a paucity of corroborating data in humans. Thus, we measured a previously validated marker for senescent cell burden in humans, T‐cell expression of *p16* mRNA, along with additional biomarkers, to compare the senescence phenotypes of postmenopausal control (lean, *N* = 37) and T2DM (*N* = 27) participants. To control for effects of obesity alone, we included a third group of obese but non‐diabetic women (*N* = 29) who were matched for body mass index to the T2DM participants. In addition, given the increase in fracture risk in T2DM despite preserved or even increased bone mineral density, we related these senescence biomarkers in the T2DM participants to skeletal microarchitectural parameters. Relative to the lean participants, T‐cell *p16* and *p21* ^*Cip1*^ expression was increased in the T2DM, but not the obese, non‐diabetic participants. Expression of *p16* and *p21* ^*Cip1*^ was positively associated with HbA1c and an index of skin advanced glycation end‐products. T2DM was also associated with an increase in a number of SASP factors. Among participants with T2DM, women in the highest tertile for T‐cell expression of *p16* had significantly reduced tibial cortical area and thickness as compared to those in the lower two tertiles. Overall, our studies link cellular senescence to metabolic and skeletal alterations in T2DM and point to the need for further studies evaluating the role of cellular senescence in mediating skeletal fragility, as well as potentially other complications in T2DM.

## Graphical abstract

In cohorts of lean controls, obese participants without Type 2 Diabetes Mellitus (T2DM) and obese T2DM participants, we find that T‐cell expression of senescence biomarkers is altered only in the T2DM participants and not with obesity alone. Moreover, high T‐cell p16 expression in the T2DM participants is associated with impaired bone cortical indices.

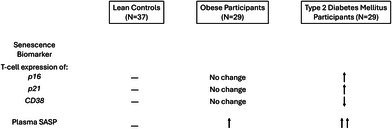

## 1 Introduction

Cellular senescence is a cell fate characterized by growth arrest, upregulation of the cyclin‐dependent kinase inhibitors, p16^Ink4a^ and p21^Cip1^, resistance to apoptosis, altered chromatin structure, and secretion of the inflammatory senescence‐associated secretory phenotype (SASP) (Khosla et al. 2022; Tchkonia et al. 2013). It is increasingly recognized as contributing to a number of age‐associated morbidities, including type 2 diabetes mellitus (T2DM) and osteoporosis (Khosla et al. 2020), although the bulk of the evidence for this comes from studies in mice. Thus, despite substantial animal data, there is a paucity of human data linking cellular senescence to T2DM or its complications. Moreover, because T2DM is typically associated with both obesity and abnormal glucose metabolism, it has been difficult to dissect the relationship of obesity per se versus T2DM to cellular senescence.

In the present study, we used a previously validated marker for senescent cell burden in humans, T‐cell expression of *p16* mRNA levels (Liu et al. 2009), along with additional biomarkers of senescence, to compare the senescence phenotypes of control (lean) and T2DM participants. In order to control for the effects of obesity, we also included a third group of carefully screened obese but non‐diabetic participants who were closely matched for BMI to the T2DM participants. We relied on T‐cell expression of *p16* to assess senescent cell burden in our study participants based on previous studies measuring *p16* ^*Ink4a*^ mRNA expression in peripheral blood T‐cells that not only found an expected age‐related increase, but *p16* ^*Ink4a*^ expression was also associated with gerontogenic behaviors such as smoking and physical inactivity (Liu et al. 2009). Additional studies found that T‐cell *p16* ^*Ink4a*^ expression was associated with plasma interleukin‐6 (IL‐6) levels (a SASP factor) (Liu et al. 2009), increased following chemotherapy (along with increases in the SASP factors MCP1 and VEGFA) (Sanoff et al. 2014), and predicted length of hospital stay after coronary artery bypass surgery in older adults (Pustavoitau et al. 2016).

As detailed in our previous studies (Farr et al. 2024, 2025), we have evaluated two relevant variants of *p16* mRNA in T‐cells: the standard *p16_variant 1* + *5* (as it is problematic to design polymerase chain reaction primers for *p16_variant 1* alone), as well as *p16_variant 5*. The dominant transcript, *p16_variant 1*, produces the established and widely studied p16^Ink4a^ protein. In comparison, *p16_variant 5*, which is expressed at much lower levels, produces the p16ɣ protein, which is identical to p16^Ink4a^ from amino acids 1–152, but has a unique 15 amino acid C‐terminal sequence that replaces the 4 amino acid C‐terminal sequence of p16^Ink4a^ (Lin et al. 2007). Importantly, although p16ɣ is produced at much lower levels than p16^Ink4a^, both proteins appear to have identical functional properties, at least in vitro (Lin et al. 2007). However, in our previous work, we found that *p16_variant 5* was more closely associated with aging in older women (Farr et al. 2024, 2025) and was more predictive of skeletal responses to a senolytic intervention (intermittent dasatinib + quercetin) than was assessment of *p16_variant 1* + *5* (Farr et al. 2024). As such, in the present study, we assessed the potential utility of both *p16* variants in evaluating senescent cell burden in the context of T2DM.

In addition, given the now well‐recognized increase in fracture risk in individuals with T2DM (Khosla et al. 2021), we related senescence biomarkers in the T2DM participants to skeletal parameters. Because postmenopausal women with T2DM are at greatest fracture risk (Khosla et al. 2021), in this initial study, we focused on this group. Moreover, previous work from our laboratory in a mouse model of adult onset T2DM demonstrated an increased senescence/SASP markers in bone as well as reductions in cortical, but not trabecular, parameters (Eckhardt et al. 2020), consistent with previous studies in T2DM participants using high resolution‐peripheral quantitative computed tomography (HR‐pQCT) [for review, see (Khosla et al. 2021)]. As such, we also explored the relationship of markers of senescent cell burden with cortical and trabecular parameters as assessed by HR‐pQCT in participants with T2DM. It is also important to note that patients with T2DM sustain fractures despite having higher bone mineral density than non‐diabetic individuals (Schwartz et al. 2011), consistent with abnormalities in “bone quality” in T2DM—e.g., impaired bone material properties related perhaps to an accumulation of advanced glycation end‐products (AGEs) in bone contributing significantly to fracture risk in these individuals (Khosla et al. 2021). As such, in terms of bone mass and structure, it is important to compare these parameters not only between control and T2DM participants but also within the T2DM participants, as these relative deficits, in the setting of impaired bone quality, likely place individuals with T2DM who have these deficits at the highest fracture risk.

## 2 Methods

### 2.1 Study Design

This was a single‐center cross‐sectional observational study of post‐menopausal women with and without obesity and/or T2DM performed at the Mayo Clinic Rochester in Minnesota, USA. Subjects were recruited between September 2019 and November 2021. The study was approved by the Mayo Clinic Institutional Review Board, and written informed consent was obtained from all participants.

### 2.2 Study Participants

Study participants were recruited by flyers, classified advertising, radio advertising, or invited to participate if they were involved in previous research. 300 subjects were screened, and we enrolled 37, 29, and 27 in the lean, obese, non‐diabetic, and T2DM groups, respectively. Participants were eligible if they were able and willing to provide informed consent, were post‐menopausal women (by history and/or follicular stimulating hormone (FSH) ≥ 16 U/L), aged 50–80 years, and had body mass index (BMI) and HbA1c as specified: (1) Lean control subjects: BMI < 25, HbA1c < 5.9%, (2) Obese, non‐diabetic subjects: BMI ≥ 30, HbA1c < 5.9%, or (3) Obese, T2DM subjects: BMI ≥ 30, HbA1c > 6.5% (for at least the past 5 years). Exclusion criteria were as follows: history of fracture prior to screening, significant liver or renal disease, malignancy (including myeloma), malabsorption, hypoparathyroidism, hyperparathyroidism, acromegaly, Cushing's syndrome, hypopituitarism, severe chronic obstructive pulmonary disease, history of cardiac failure, bleeding disorders, or current use of therapeutic doses of anticoagulants other than aspirin, or if they were on medications that affect bone turnover, such as corticosteroids > 3 months at any time (inhaled steroids acceptable unless used year‐round), anticonvulsant therapy for seizures within the past year, thiazolidinediones, thyroid hormone causing a decline of TSH below normal, bisphosphonates within the past 3 years, denosumab, estrogen therapy, or selective estrogen receptor modulator within the last year, or if they had clinically significant abnormal screening laboratory studies within the past 3 months: complete blood count (CBC), estimated glomerular filtration rate (eGFR) < 30 mL/min, alanine transaminase (AST) > 2‐fold elevated, calcium > 10.5 mg/dL.

### 2.3 Measurement of T‐Cell mRNA Levels

Morning fasting peripheral whole blood was obtained from the study participants for measuring *p16_variant 1* + *5*, *p16_variant 5*, *p21* ^*Cip1*^, and *CD28* mRNA levels in CD3^+^ peripheral blood T‐cells by reverse transcriptase‐quantitative polymerase chain reaction (rt‐qPCR), as previously described (Farr et al. 2024, 2025). Table S1 provides the primers used for these assays.

### 2.4 Measurement of Plasma SASP Proteins

SASP proteins were measured in morning fasting plasma samples using commercially available multiplex magnetic bead immunoassays (R&D Systems) based on a Luminex xMAP multianalyte profiling platform. These assays were performed according to the manufacturer's protocols and analyzed on a MAGPIX System (Merck Millipore), as described previously (Schafer et al. 2020). An exception was activin A, which was measured by a Quantikine ELISA Kit (R&D Systems).

### 2.5 Skin AGEs

Skin AGEs were measured using the AGE Reader (Diagnoptics), which measures an index of tissue accumulation of AGEs by means of skin autofluorescence (Meerwaldt et al. 2004) using an excitation light source with a wavelength between 300 and 420 nm. Previous studies have shown an error rate of ~5% when repeated skin autofluorescence measurements are made in control and T2DM participants (Meerwaldt et al. 2004).

### 2.6 Dual‐Energy X‐Ray Absorptiometry (DXA)

Areal BMD measurements at the hip, L1‐L4 antero‐posterior spine, and forearm were made with DXA (Lunar iDXA; GE Medical Systems).

### 2.7 Trabecular Bone Score (TBS)

The lumbar spine images obtained for the DXA were used to calculate a trabecular bone score using TBSiNsight software (TBS iNsight, Medimaps, Merignac, France).

### 2.8 High‐Resolution Peripheral Quantitative Computed Tomography (HRpQCT)

We used a validated protocol (Scanco Medical AG) to obtain high‐resolution in vivo images of the distal radius and tibia with the XtremeCT, as previously described (Nicks et al. 2012), except we used the newest generation scanner, XtremeCT II, which offers higher resolution (61 μm versus 82 μm on the XtremeCT I). Further, we performed additional HRpQCT measurements at more proximal sites at both the radius and tibia to better assess cortical bone parameters (Patsch et al. 2013). The standard scan regions are referred to as “ultradistal,” while the more proximal regions are referred to as “distal” region. The non‐dominant forearm and ankle were scanned, except in subjects with a prior local fracture, in whom the non‐fractured side was scanned. Image quality was graded by a trained technician according to the manufacturer‐suggested image grading system (grade 1 [no visible motion artifacts] to grade 5 [severe motion artifacts]). Scans were repeated for images with a score of 4 or 5. Trabecular bone volume fraction (Tb.BV/TV), trabecular number, trabecular thickness, and trabecular separation were derived as previously described (Nicks et al. 2012). For the cortical parameters, the extended cortical analysis for XtremeCT II was used to obtain intra‐cortical porosity, cortical thickness, cortical volumetric BMD (vBMD), cortical area, and cortical periosteal perimeter. The validity of these approaches has been rigorously tested, and excellent correlations (*r* ≥ 0.96) were shown between HRpQCT and the “gold standard” ex vivo micro‐CT technique (MacNeil and Boyd 2007). In addition, linear micro‐finite element (μFE) models of the distal radius and tibia were created directly from the HRpQCT images to assess in vivo indices of biomechanical bone strength such as stiffness and failure load, as previously described (Farr et al. 2012). Quality control results were tracked using standard control charts. Potential problems (e.g., drift of the phantom measurements) triggered closer examination of the machines and, if appropriate, measurements made during the suspect time were adjusted. Daily QC scans for the XtremeCT II use protocol settings for the radius.

### 2.9 Statistical Analyses

Continuous variables were summarized using median and inter‐quartile range (IQR, Q1–Q3), and categorical variables were summarized using count (%). Group differences were assessed using the Kruskal–Wallis test for continuous variables and the chi‐squared test for categorical variables. *p*‐values for pairwise comparisons between the three groups were adjusted using the Holm method. Spearman correlation coefficients were used to measure the relationship between select continuous variables. A two‐tailed probability value of *p* < 0.05 was considered statistically significant for all tests. Statistical analysis was conducted using R version 4.4.1.

## 3 Results

### 3.1 Baseline Characteristics of Study Participants

Table 1 shows the baseline parameters in the three groups of study participants (lean, obese, and T2DM). All participants were post‐menopausal females, and the majority (96.8%) were white. There were no differences in demographics between the lean, obese, and T2DM groups, such as age, race, ethnicity, or height. BMI was significantly higher in the obese and T2DM groups at 36.5 and 36.3 kg/m^2^, respectively, compared to 22.4 kg/m^2^ in the lean group. Importantly, the obese and T2DM groups had similar BMI values but differed significantly in HbA1c values (7.6% vs. 5.5% in the T2DM and obese groups, respectively), allowing us to clearly separate the effects of obesity per se versus obesity plus T2DM on our outcome variables. Skin AGEs were significantly higher in the T2DM group as compared to either the lean or the obese groups. In T2DM participants, the median duration of diabetes was 15 (12–22) years, with 92.6% diagnosed more than 10 years ago. The majority (88.9%) were on metformin, 44.4% were on a glucagon‐like peptide 1 receptor (GLP1R) agonist, and 51.9% were on insulin.

**TABLE 1:** Baseline characteristics of study participants.

	Lean (*N* = 37)	Obese (*N* = 29)	T2DM (*N* = 27)
Demographics			
Age (years)	70.1 (63.0–72.2)	64.7 (59.8–73.7)	66.7 (62.8–71.4)
Female, *n* (%)	37 (100.0%)	29 (100.0%)	27 (100.0%)
Post‐menopausal	37 (100.0%)	29 (100.0%)	27 (100.0%)
Natural menopause	30 (81.1%)	20 (69.0%)	21 (77.8%)
Race			
White	36 (97.3%)	28 (96.6%)	26 (96.3%)
American Indian	0 (0.0%)	1 (3.4%)	0 (0.0%)
Asian	1 (2.7%)	0 (0.0%)	1 (3.7%)
BMI (kg/m^2^)	22.7 (21.6–23.7)	34.7 (32.0–40.0)***	36.6 (34.1–38.5)***
Height (cm)	162.7 (159.1–167.6)	160.7 (158.6–166.9)	162.1 (159.9–166.8)
Weight (kg)	60.9 (52.3–64.8)	89.1 (83.1–106.7)***	97.9 (88.1–101.5)***
Percentage fat mass (%)	35.3 (30.5–38.7)	47.6 (45.1–51.6)***	46.3 (42.9–48.5)***
Percentage lean mass (%)	61.3 (57.7–66.1)	49.6 (45.9–52.0)***	51.3 (48.8–54.3)***,†
Duration of diabetes	—	—	15 (12–21)
Diabetes medications, *n* (%)	—	—	
Metformin			24 (88.9)
Sulphonylurea			6 (22.2)
GLP1R‐agonist			12 (44.4)
Insulin			14 (51.9)
HbA1c (%)	5.4 (5.2–5.6)	5.5 (5.4–5.6)	7.6 (7.2–8.0)***,†††
25(OH)D, ng/mL	39 (33–50)	40 (29–52)	37 (28.5–45.5)
Skin AGEs	2.2 (2.0–2.4)	2.3 (2.0–2.6)	2.8 (2.5–3.3)***,†††

*Note:* Bold text highlights significantly different values among groups. ***p < 0.001 compared to the Lean; †*p* < 0.05, †††*p* < 0.001 for comparison of T2DM vs. Obese.

### 3.2 Expression of Senescence Markers in T‐Cells

**FIGURE 1:**
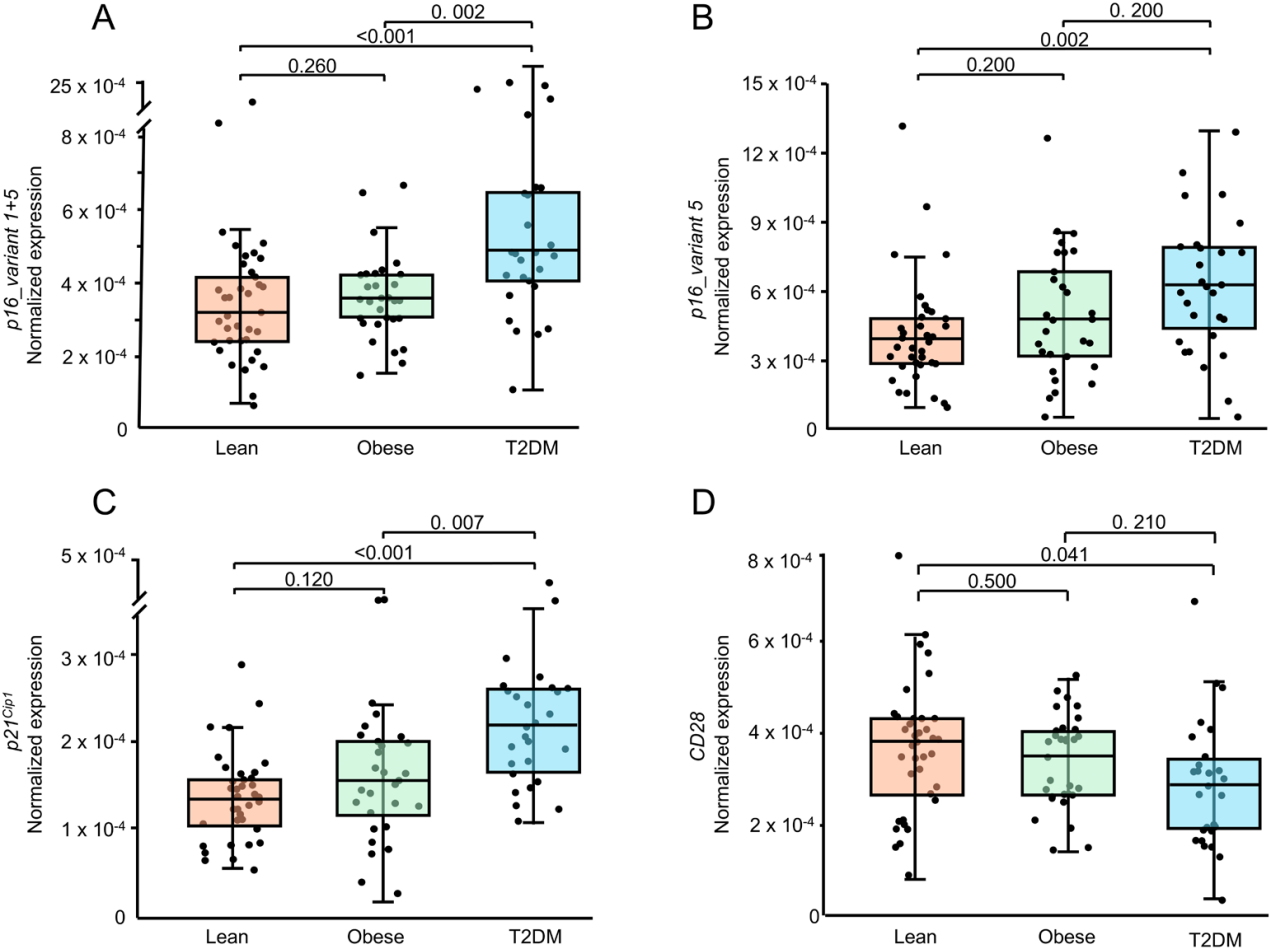
Normalized mRNA expression in T‐cells of (A) *p16_variant 1* + *5*, (B) *p16_variant 5*, (C) *p21* ^*Cip1*^, and (D) *CD28* in the study groups.

As shown in Figure 1A, expression of *p16_variant 1* + *5* was significantly higher in the T2DM participants compared to either the lean or obese participants. Expression of *p16_variant 5* (Figure 1B) was also significantly higher in the T2DM participants compared to the lean participants, but was not significantly different in the T2DM group compared to the obese group. Thus, *p16_variant 1* + *5* appeared to better distinguish the T2DM participants than *p16_variant 5*, not only from the lean controls but also from the obese, non‐diabetic participants. Expression of neither *p16_variant 1* + *5 n*or *p16_variant 5* was different between the obese and the lean control participants. Expression of *p21* ^*Cip1*^ (Figure 1C) showed a generally similar pattern, with T2DM participants expressing higher levels than either the lean control or obese participants. Using carefully matched obese and T2DM participants, these findings thus demonstrate that expression of the key senescence markers, *p16* and *p21* ^*Cip*^, in peripheral T‐cells is increased specifically in T2DM but not in obesity alone. Interestingly, reduction and/or loss of expression of *CD28* has been associated with T‐cell aging/senescence (Pangrazzi and Weinberger 2020; Vallejo 2005), and *CD28* expression was significantly lower in the T2DM group compared to the lean controls (Figure 1D).

Since both metformin (Kim, Yu et al. 2021; Noren Hooten et al. 2016) and GLP1R‐agonists (Zhang et al. 2019) have been associated with possible effects on senescence and aging, we also examined whether T2DM participants on these drugs differed in terms of senescence or other markers as compared to those not taking them. This was only possible for the GLP1R‐agonists, as there were only three participants in our cohort who were not on metformin (Table 1). As shown in Table S2, none of the senescence markers from Figure 1 or HbA1c/skin AGEs differed significantly between GLP1R‐agonist users as compared to non‐users.

We next examined relationships between the *p16* variants, *p21* ^*Cip1*^, and metabolic parameters (HbA1c and skin AGEs) in all participants combined (Table 2). T‐cell expression of *p16_variant 1* + *5* was positively associated with expression of *p16_variant 5*, *p21* ^*Cip1*^, HbA1c, and skin AGEs, and negatively associated with expression of *CD28*. *p16_variant 5* showed generally similar, but weaker, correlations and was not significantly associated with HbA1c levels. Similar associations were found for *p21* ^*Cip1*^, although it was not associated with *CD28* expression. As expected, HbA1c was strongly associated with skin AGE levels. Overall, these data support that T‐cell expression of *p16_variant 1* + *5*, in particular, is associated with other markers of cellular senescence in T‐cells, as well as with metabolic parameters (HbA1c and skin AGEs).

**TABLE 2:** Correlation matrix showing *R*‐ and *p*‐values (Spearman correlations) between the T‐cell senescence markers, HbA1c, and Skin AGEs.

	*p16_variant 1* + *5*	*p16_variant 5*	*p21* ^*Cip1*^	*CD28*	HbA1c	Skin AGEs
*p16_variant 1* + *5*	—	0.41***	0.27**	−0.34**	0.32**	0.33**
*p16_variant 5*	0.41***	—	0.29**	−0.21*	0.20	0.25*
*p21* ^*Cip1*^	0.27**	0.29**	—	0.03	0.31**	0.37***
*CD28*	−0.34**	−0.021*	0.03	—	−0.21*	−0.17
HbA1c	0.32**	0.20	0.31**	−0.21*		0.51***
Skin AGEs	0.33**	0.25*	0.37***	−0.17	0.51***	—

*Note:* Bold text highlights statistically significant correlations. *p < 0.05; **p < 0.01; ***p < 0.001.

### 3.3 Circulating SASP Factors

Table 3 shows the plasma concentrations of 36 SASP factors by group. Concentrations of activin‐A, Fas, IL‐6, TNFR1, and TNFR2 were higher in both obese and T2DM participants than in lean participants, and concentrations of GDF‐15, GROa, IL‐8, and TNFa were also higher in participants with T2DM compared to lean controls. Moreover, GDF15 and IL‐8 were significantly higher in T2DM than in obese participants.

**TABLE 3:** Circulating SASP factors in the study participants. All units are in pg/mL.

SASP factors	Lean (*n* = 37)	Obese (*n* = 29)	T2DM (*n* = 27)
Activin‐A	273 (240–309)	339 (269–366)**	346 (273–439)*
ADAMTS13 (×10^4^)	98.1 (68.1–116.8)	84.7 (61.4–121.0)	90.0 (72.1–114.5)
Eotaxin	263 (206–337)	232 (170–493)	288 (218–369)
Fas (×10^3^)	7.66 (6.20–8.95)	8.86 (7.95–10.30)*	9.41 (8.31–10.96)***
GDF‐15	583 (478–793)	612 (553–796)	1244 (883–1662)***,†††
GROα	136 (121–150)	150 (121–175)	163 (136–186)**
ICAM‐1 (×10^5^)	2.35 (2.02–2.76)	2.57 (2.01–3.07)	2.43 (2.14–3.07)
IFN‐*γ*	0.98 (0.65–1.25)	0.65 (0.51–1.15)	0.95 (0.75–1.37)
IL‐6	1.71 (1.32–2.16)	2.98 (2.51–4.02)***	3.85 (2.85–4.56)***
IL‐7	18.4 (15.4–23.4)	20.5 (15.4–24.5)	16.2 (14.0–21.6)
IL‐8	12.1 (10.5–15.5)	12.5 (10.7–18.2)	16.6 (14.3–22.7)**,†
IL‐15	2.12 (1.86–2.65)	2.19 (1.97–2.60)	2.26 (1.90–2.71)
MCP‐1	405 (340–457)	354 (305–434)	374 (322–525)
MDC	324 (264–489)	340 (280–521)	397 (319–655)
MMP‐1 (×10^3^)	4.18 (2.73–5.33)	3.51 (2.23–5.00)	3.53 (2.27–5.03)
MMP‐2 (×10^5^)	2.61 (2.22–3.05)	2.48 (2.20–2.71)	2.60 (2.08–2.80)
MMP‐7 (×10^2^)	16.8 (14.8–33.3)	26.4 (20.7–31.0)	30.3 (24.4–38.0)
MMP‐9	13.23 (11.68–21.81)	16.68 (12.66–22.42)	19.10 (13.71–26.77)
MPO (×10^5^)	1.40 (0.96–1.97)	1.79 (0.99–2.73)	1.58 (0.79–2.60)
Osteoactivin (×10^3^)	16.4 (14.6–20.8)	18.5 (16.3–20.9)	19.5 (16.9–22.1)
Osteopontin (×10^3^)	8.50 (4.77–16.00)	9.96 (5.60–16.50)	8.76 (2.52–12.08)
PAI‐1 (×10^5^)	1.21 (0.99–1.52)	1.17 (0.93–1.60)	1.44 (1.14–1.66)
PARC (×10^4^)	5.25 (4.24–6.54)	6.26 (4.66–8.10)	5.45 (4.54–7.46)
PDGF‐AA (×10^3^)	13.4 (8.3–28.1)	11.9 (7.8–28.1)	10.7 (7.5–28.1)
PDGF‐AB (×10^3^)	4.48 (3.49–5.93)	4.03 (2.83–5.63)	4.57 (3.25–5.25)
PLA2G7 (×10^4^)	3.82 (3.08–4.94)	4.68 (3.77–6.11)	4.70 (3.84–5.53)
RAGE (×10^3^)	3.55 (2.25–4.36)	3.41 (2.73–4.02)	3.10 (2.23–3.52)
RANTES (×10^4^)	4.50 (3.70–5.64)	4.17 (3.03–6.32)	4.71 (3.70–6.51)
SOST	181 (114–284)	194 (135–263)	212 (132–324)
SPARC (×10^6^)	1.67 (1.24–2.14)	1.60 (1.41–2.07)	1.87 (1.34–2.10)
TNFα	11.4 (10.0–12.7)	12.1 (10.2–15.0)	14.2 (12.9–16.2)***
TNFR1 (×10^3^)	1.15 (0.92–1.28)	1.38 (1.23–1.68)**	1.56 (1.26–1.98)***
TNFR2 (×10^3^)	2.52 (1.89–3.05)	2.97 (2.58–3.74)*	3.42 (2.37–4.33)*
TRAIL	88.9 (69.5–100.4)	88.0 (71.5–106.7)	81.8 (17.5–99.4)
uPAR (×10^3^)	1.12 (0.75–1.42)	1.22 (0.86–1.52)	1.35 (1.09–1.77)
VEGF	231 (158–364)	284 (194–399)	319 (217–440)

*Note:* Bold text highlights significantly different values among groups. *p < 0.05, **p < 0.01, and ***p < 0.001 compared to the Lean; †*p* < 0.05, †††*p* < 0.001 for the comparison of T2DM vs. Obese.

### 3.4 DXA and HRpQCT Skeletal Parameters in the 3 Groups

Table 4 shows the DXA and HRpQCT parameters in the 3 groups. Overall, both the obese and T2DM participants had better values for these parameters compared to the lean participants, consistent with obesity itself being associated with better skeletal mass and structure (Evans et al. 2015).

**TABLE 4:** Skeletal parameters as measured by DXA and HRpQCT in the 3 groups.

	Lean (*N* = 37)	Obese (*N* = 29)	T2DM (*N* = 27)
DXA BMD			
Femur Neck (g/cm^2^)	0.814 (0.747–0.917)	0.901 (0.845–0.955)*	0.954 (0.822–1.073)***
Total Femur (g/cm^2^)	0.843 (0.776–0.952)	0.971 (0.925–1.064)***	1.045 (0.969–1.184)***,†
Spine‐L1–L4 (g/cm^2^)	1.08 (0.97–1.15)	1.26 (1.13–1.33)***	1.23 (1.13–1.51)***
Spine‐L1–L4 TBS	1.31 (1.28–1.36)	1.41 (1.35–1.52)***	1.36 (1.28–1.46)
Forearm radius UD (g/cm^2^)	0.332 (0.286–0.356)	0.468 (0.406–0.509)***	0.455 (0.425–0.503)***
Forearm total radius (g/cm^2^)	0.511 (0.467–0.600)	0.689 (0.621–0.731)***	0.672 (0.644–0.725)***
HRpQCT Ultradistal Radius			
Trabecular BV/TV	0.184 (0.149–0.210)	0.220 (0.174–0.245)*	0.225 (0.208–0.269)***
Trabecular number (1/mm)	1.30 (1.14–1.45)	1.48 (1.35–1.60)*	1.58 (1.49–1.73)***
Trabecular thickness (mm)	0.229 (0.223–0.236)	0.231 (0.223–0.241)	0.231 (0.225–0.243)
Trabecular separation (mm)	0.775 (0.672–0.878)	0.637 (0.589–0.733)*	0.601 (0.560–0.675)***
Stiffness (kN/mm)	44.2 (36.9–49.9)	53.6 (46.6–69.4)**	59.3 (49.0–66.7)***
Failure load (kN)	2.39 (2.02–2.72)	2.92 (2.52–3.73)**	3.29 (2.69–3.74)***
HRpQCT distal radius			
Cortical vBMD (mg HA/cm^3^)	992 (964–1032)	1015 (999–1042)	1013 (970–1043)
Cortical area (mm^2^)	61.6 (57.5, 69.3)	73.9 (67.6–78.4)***	75.0 (69.2–79.2)***
Cortical thickness (mm)	1.76 (1.54–1.95)	1.94 (1.77–2.06)*	2.00 (1.92–2.12)**
Periosteal perimeter (mm)	47.4 (43.9–49.1)	49.3 (46.0–52.2)	47.9 (46.0–51.1)
Intra‐cortical porosity	0.018 (0.011–0.027)	0.011 (0.008–0.016)*	0.010 (0.007–0.021)*
HRpQCT Ultradistal Tibia			
Trabecular BV/TV	0.210 (0.185–0.255)	0.251 (0.224–0.280)**	0.275 (0.236–0.298)**
Trabecular number (mm^−1^)	1.39 (1.22–1.49)	1.53 (1.39–1.67)**	1.59 (1.41–1.69)**
Trabecular thickness (mm)	0.253 (0.243–0.261)	0.253 (0.245–0.268)	0.277 (0.249–0.282)
Trabecular separation (mm)	0.721 (0.668–0.828)	0.664 (0.592–0.731)*	0.638 (0.585–0.712)**
Stiffness (kN/mm)	131.5 (112.7–148.8)	169.6 (159.1–181.3)***	176.7 (161.2–187.3)***
Failure load (kN)	7.25 (6.36–8.04)	9.25 (8.81–9.96)***	9.65 (8.79–10.27)***
HRpQCT Distal Tibia			
Cortical vBMD (mg HA/cm^3^)	912 (857–954)	933 (904–947)	936 (886–969)
Cortical area (mm^2^)	139 (129–154)	166 (157–173)***	168 (160–180)***
Cortical thickness (mm)	2.08 (1.77–2.32)	2.33 (2.20–2.56)**	2.45 (2.29–2.66)***
Periosteal perimeter (mm)	83.2 (77.4–86.6)	84.6 (80.9–91.2)	85.6 (82.8–87.9)
Intra‐cortical porosity	0.037 (0.026–0.042)	0.034 (0.027–0.043)	0.030 (0.023–0.033)

*Note:* Bold text highlights significantly different values among groups. *p < 0.05, **p < 0.01, and ***p < 0.001 compared to the Lean; †*p* < 0.05 for comparison of T2DM vs. Obese.

In the context of T2DM and senescence, we next posed the question of whether relative skeletal deficits within the T2DM participants were related to T‐cell *p16* mRNA levels. As noted earlier, against the background of impaired bone material properties in T2DM, it is likely that these relative deficits within the T2DM group contribute to an increase in fracture risk (Khosla et al. 2021). In this analysis, we focused on T‐cell *p16* mRNA levels, as our previous study had found that stratification of postmenopausal women based on this measure was predictive of the skeletal response to a senolytic intervention (dasatinib + quercetin) (Farr et al. 2024). To test this, we used the *p16_variant 1* + *5* expression, as this variant appeared to demonstrate better separation from both the lean and obese controls as compared to *p16_variant 5* expression (Figure 1A,B) and also correlated more strongly with metabolic parameters (Table 2).

**FIGURE 2:**
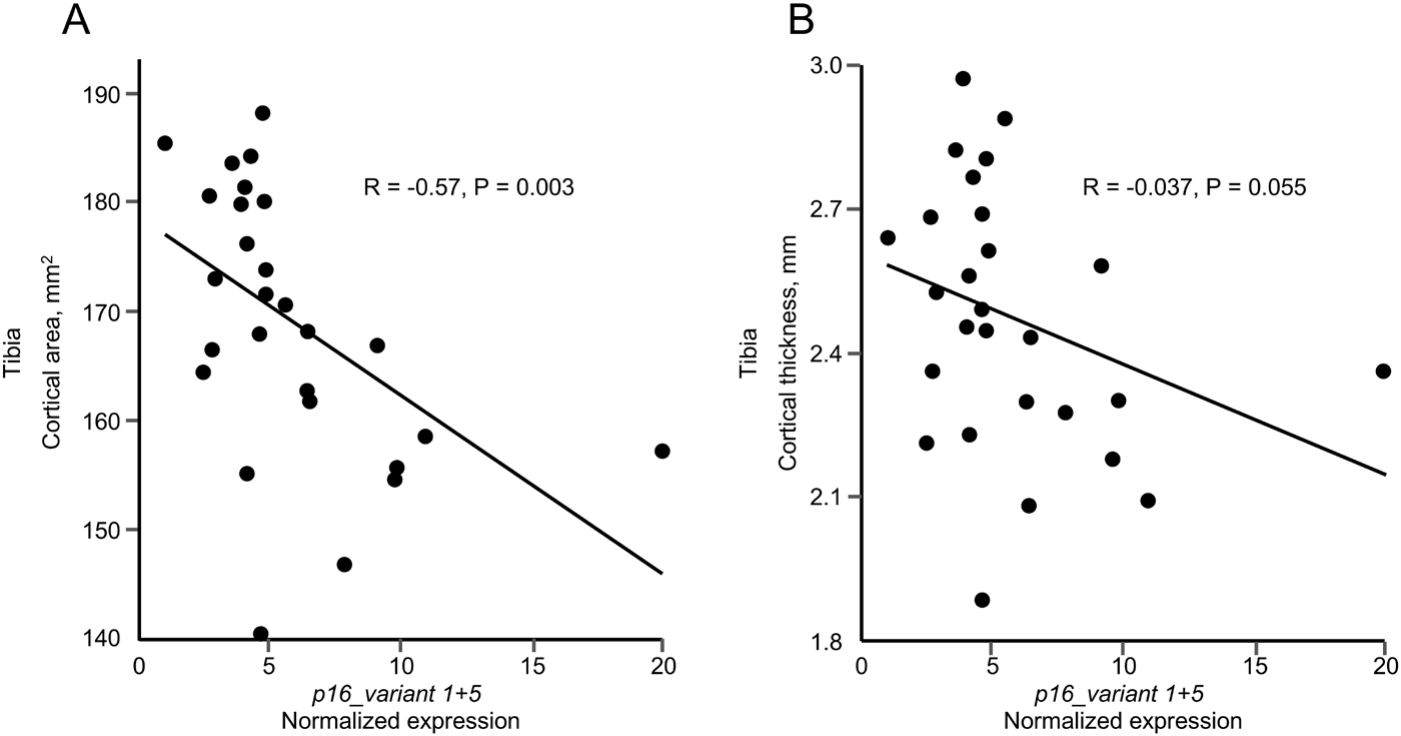
Correlation of tibial (A) cortical area and (B) cortical thickness with *p16_variant 1* + *5* expression in T‐cells in T2DM participants. Correlations and *p*‐values are using non‐parametric Spearman correlations.

We initially stratified the T2DM participants, as we did previously in the context of aging (Farr et al. 2024, 2025), into tertiles of *p16_variant 1* + *5* expression and compared the skeletal indices across tertiles (T1, T2, T3). As shown in Table S3, none of the trabecular parameters at the radius or tibia (with the exception of an inconsistent association [T2 being higher than T1 or T3] for trabecular thickness at the tibia) differed across the *p16_variant 1* + *5* tertiles. By contrast, both cortical area and thickness at the tibia showed a clear trend (T3 lower than T1 or T2) across tertiles. To explore this further, we combined the lower 2 tertiles (T1/T2) of *p16_variant 1* + *5* expression and compared T3 to this combined group—again, similar to our previous approach in the context of aging (Farr et al. 2024, 2025) (Table 5). In this analysis, both cortical area and thickness were significantly lower in the T3 subset of T2DM participants as compared to the T1/T2 group. Moreover, in the combined group of T2DM participants, both cortical area (Figure 2A) and cortical thickness (Figure 2B) at the distal tibia were negatively correlated with T‐cell *p16_variant 1* + *5* mRNA levels. Thus, senescent cell burden in T2DM, at least as assessed by expression of T‐cell *p16_variant 1* + *5* mRNA levels, appears to largely spare trabecular bone and predominantly have detrimental effects on cortical bone. We note that in exploratory analyses, the SASP factors that were distinctly higher in T2DM than obese/non‐diabetic participants (GDF‐15 and IL‐8) did not demonstrate significant associations with measures of cortical bone (*R* = 0.17 and 0.09 for GDF15 and *R* = −0.02 and 0.01 for IL‐8 for tibial cortical area and thickness, respectively, all *p* > 0.05).

**TABLE 5:** Cortical parameters at the distal tibia by HRpQCT in the T2DM participants stratified by tertiles (tertile 1 and 2, T1/T2 vs. tertile 3, T3) for expression of T‐cell *p16_variant 1* + *5* mRNA levels.

	T1/T2	T3	*p*
Cortical vBMD (mg HA/cm^3^)	931 (888, 963)	937 (880, 984)	0.572
Cortical area (mm^2^)	**175 (169, 181)**	**159 (156, 163)**	**0.002**
Cortical thickness (mm)	**2.59 (2.45, 2.74)**	**2.30 (2.18, 2.36)**	**0.007**
Periosteal perimeter (mm)	86.3 (83.6, 88.0)	84.8 (81.7, 87.2)	0.681
Intra‐cortical porosity	0.031 (0.024, 0.041)	0.027 (0.022, 0.031)	0.135

*Note:* Bold text highlights significantly different values among groups.

## 4 Discussion

Using carefully selected lean, obese, and T2DM participants, we demonstrate in the present study that obesity alone (in the absence of T2DM) does not result in an increase in T‐cell *p16* mRNA (either *variant 1* + *5* or *variant 5*) or *p21* ^*Cip1*^ expression. Moreover, although the obese participants demonstrated an increase in some SASP factors, a larger number of the SASP factors were increased in the T2DM participants. Thus, senescent cell burden, at least as assessed by these biomarkers, was increased principally in the T2DM participants. These findings are entirely consistent with our previous findings in mice (Eckhardt et al. 2020), where we demonstrated increases in *p16* ^*Ink4a*^ (note that there is no *p16_variant 5* in mice (Farr et al. 2025)) as well as *p21* ^*Cip1*^ expression in osteocyte‐enriched bone samples from mice with adult‐onset T2DM as compared to lean control mice. Those findings were further corroborated by demonstrating increases in more specific measures of senescence—i.e., senescence‐associated distension of satellites (SADS) as well as in telomere‐associated foci (TAF) in osteocytes in the T2DM mice (Eckhardt et al. 2020). Collectively, these animal and human data indicate that although obesity itself is likely associated with an inflammatory state, as reflected by an increase in some SASP factors, evidence for an increase in senescent cell burden, at least as measured by the biomarkers used here, is present only with the development of T2DM.

We also found that T‐cell expression of both *p16_variant 1* + *5* and *p21* ^*Cip1*^ was associated with HbA1c and with skin AGEs, further linking the metabolic abnormalities in T2DM to cellular senescence. Of interest, T‐cell expression of *CD28*, which is lost with the onset of T‐cell exhaustion/senescence (Pangrazzi and Weinberger 2020; Vallejo 2005), was also reduced in the T2DM participants and showed an inverse association with expression of both *p16* variants in T‐cells. Thus, although whether T‐cells truly develop a fully senescent phenotype as conventionally defined for mesenchymal cells remains open to debate (Slaets et al. 2024), it does appear that T‐cell expression of *p16* is linked to the loss of a key marker associated with T‐cell function, *CD28* (Pangrazzi and Weinberger 2020; Vallejo 2005).

In terms of the effects of T2DM medications on cellular senescence, drugs such as metformin and sodium‐glucose cotransporter‐2 inhibitors (SGLT2i) have shown promising anti‐aging effects in preclinical studies, particularly in ameliorating cellular senescence in various tissues ranging from mouse mandible to human adipocytes and renal tubular epithelial cells (Eleftheriadis et al. 2022; Katsuumi et al. 2024; Kim, Yu et al. 2021; Kim, Moon, and Cho 2021; Liu et al. 2024; Noren Hooten et al. 2016). However, the increase in T‐cell *p16* and *p21* ^*Cip1*^ expression in our T2DM cohort despite concurrent use of metformin further supports the association of metabolic abnormalities in T2DM to cellular senescence. None of our patients were on SGLT2i; hence, this is unlikely to be confounding. In the literature, GLP1R‐agonists have had more variable effects on cellular senescence—liraglutide induced cellular senescence and had an anti‐proliferative effect in hepatocellular carcinoma cells (Krause et al. 2017), while exendin‐4 downregulated the expression of *p16* and *p21* ^*Cip1*^ in rat osteoblasts (Zhang et al. 2019). In our study, subgroup analysis of patients on GLP1R‐agonists compared to those not on these drugs did not reveal any discernible difference in the senescence markers *p16*, *p21* ^*Cip1*^, or *CD28*. While GLP1R‐agonists have demonstrated a protective effect on bone in animal models (Ma et al. 2013; Pereira et al. 2015), this may be mediated through alternative mechanisms independent of cellular senescence (Zhang et al. 2019), and clinical studies thus far have not supported a beneficial impact of these drugs on bone health (Driessen et al. 2015; Mabilleau et al. 2014). Additional human studies on the impact of T2DM medications on cellular senescence and bone fragility are needed to further investigate any potential effect of these medications on bone health in T2DM.

In addition to linking cellular senescence to metabolic changes, given that fracture risk is increased in T2DM (Khosla et al. 2021), we also explored the possible relationship between senescence and skeletal mass and structure in these participants. Note that because we were interested in identifying underlying mechanisms, we excluded participants with a history of fracture from the lean and obese controls. For consistency, we also excluded T2DM participants with a fracture history, which would perhaps represent a conservative bias in our findings, as presumably T2DM participants with a fracture history may have had even more substantial alterations in the senescence biomarkers. With this caveat in mind, we found that the obese and T2DM participants generally had better measures of skeletal mass and structure than the lean controls, consistent with a protective effect of obesity on the skeleton (Evans et al. 2015).

It has become clear, however, that patients with T2DM sustain fractures at higher levels of bone mineral density (Schwartz et al. 2011) and this is likely due to the impaired bone quality in these patients, perhaps due to excessive skeletal accumulation of AGEs (Khosla et al. 2021). Although we did not directly assess bone AGEs, skin AGEs were higher in the T2DM participants as compared to either the lean or obese controls, and we previously found that skin AGEs correlate with bone material properties in patients with T2DM as directly assessed by microindentation (Samakkarnthai et al. 2020). Thus, in the setting of impaired bone quality, additional structural deficits within the T2DM participants may identify the subset of diabetic individuals at increased fracture risk. In order to test for any links between possible skeletal deficits and senescence, we stratified the T2DM participants based on their senescent cell burden as assessed by T‐cell *p16_variant 1* + *5* expression and found that the T2DM participants in the highest tertile had significantly reduced tibial cortical area and thickness but preserved trabecular indices. Moreover, within all T2DM participants, tibial cortical area and thickness were negatively correlated with T‐cell expression of *p16_variant 1* + *5*. In terms of the magnitude of these changes, even though cortical thickness was only ~11% lower in the T2DM participants in the highest tertile for T‐cell *p16_variant 1* + *5* expression as compared to those in the lower 2 tertiles, the effects on bending strength would be even greater, given that this is related to the cross‐sectional moment of inertia (which scales to fourth power of the radius), resulting in 20%–30% reductions in bending strength.

Our findings are entirely consistent with a recent, large study of skeletal alterations in T2DM participants, which found that those with a history of fracture had clearly reduced cortical area and thickness (Agarwal et al. 2024), similar to the T2DM participants in our study who were in the highest tertile for *p16_variant 1* + *5* expression. In addition, our findings are also consistent with our previous mouse data demonstrating that adult‐onset T2DM was associated with preserved trabecular parameters but reduced cortical volumetric BMD and thickness (Eckhardt et al. 2020). Thus, collectively the human data from our and previous studies, along with the animal data, suggest that the development of cellular senescence in T2DM leads principally to cortical deficits which are most pronounced in T2DM participants with fracture, although this latter conclusion is based not on our data but previous studies in this population (Agarwal et al. 2024).

In terms of *p16* variants, we previously found that T‐cell expression of *p16_variant 5* was more closely associated with age (Farr et al. 2024, 2025) and performed better in identifying postmenopausal women with a favorable skeletal response to a senolytic intervention (dasatinib + quercetin) than was expression of *p16_variant 1* + *5* (Farr et al. 2024). By contrast, in the context of T2DM, we now find that expression of *p16_variant 1* + *5* was better at separating the T2DM from the obese, non‐diabetic participants and was also more strongly correlated with metabolic parameters than was expression of *p16_variant 5*. These data indicate that future studies should assess both variants in different conditions associated with cellular senescence, as expression of one variant or the other may be more responsive to the specific conditions driving cellular senescence.

We acknowledge several limitations of our study. Its cross‐sectional design prevents the assessment of causal relationships between senescence burden and age‐related changes in bone parameters. Additionally, the study population consisted of community‐dwelling women with no history of fractures and well‐controlled glycemia. As a result, the findings may not be generalizable to more frail individuals, those with poorly controlled T2DM, or men. In addition, we note that our study participants were predominantly white (> 96%), with no black or Hispanic participants. Thus, additional studies are needed to extend our findings to non‐white populations with T2DM. A key strength of the study was the exclusion of many potential confounding comorbidities and medications directly affecting bone metabolism (e.g., bisphosphonates), allowing for a clearer assessment of the skeletal effects of T2DM.

In summary, our work demonstrates that T2DM is associated with an increase in senescent cell burden, at least as assessed by the specific biomarkers used in this study. Senescent cell biomarkers also correlated with metabolic parameters, and high levels of a specific biomarker, T‐cell expression of *p16_variant 1* + *5*, were associated with deficits in cortical bone in T2DM participants, which has previously been linked to increased fracture risk in these individuals (Agarwal et al. 2024). Given our findings, further studies are clearly needed to evaluate the role of cellular senescence in mediating skeletal fragility as well as potentially other complications in T2DM.

## Author Contributions

J.G.S. and S.K. conceived and directed the project, with contributions from A.K.P. A.T. recruited the study participants. C.H., J.G.S., S.K., J.N.F., and D.G.M. analyzed the data. N.K.L.'s laboratory performed the SASP analyses (with T.A.W.). E.J.A. performed the statistical analyses. C.H., J.G.S., and S.K. wrote the manuscript, which all authors then reviewed and approved.

## Ethics Statement

All studies were approved by the Mayo Clinic Institutional Review Board, and written informed consent was obtained from all participants. All human studies were approved by the Mayo Clinic Institutional Review Board.

## Conflicts of Interest

Mayo Clinic and A.K.P. have intellectual property related to this work. This research has been reviewed by the Mayo Clinic Conflicts of Interest Review Board and is being conducted in compliance with Mayo Clinic Conflicts of Interest policies.

## Supporting information


Tables S1–S3.

## Data Availability

All information on materials and reagents is provided in the Methods. Individual deidentified participant data that underlie the results reported in this article (text, tables, figures) will be made available from the corresponding author upon reasonable request.
