# Supplementary material for: Biomarkers of Cellular Senescence in Type 2 Diabetes Mellitus
Source: Aging Cell. 2025 May 30;24(8):e70120. doi: 10.1111/acel.70120 (PMC12341793; doi:10.1111/acel.70120)
Supplement: Supplementary file 1 — Tables S1–S3. [file ACEL-24-e70120-s001.pdf]

Supplementary Table 1. qPCR primer sequences used in the study.

Gene	Forward Primer Sequence	Reverse Primer Sequence
ACTB (Housekeeping)	CCCAGCCATGTACGTTGCTAT	TCACCGGAGTCCATCACGAT
CDKN2A_variant 5	CAGAAATGATCGGAAACCATT	CTACGCATGCCTGCTTCTAC
CDKN2A_variant 1+5	CCAACGCACCGAATAGTTACG	GCGCTGCCCATCATCATG
$p21^{Cip1}$	GGCAGACCAGCATGACAGATT	GGCTTCCTCTTGGAGAAGATCA
CD28	CCTATTTCCCGGACCTTCTA	ATAAAGGCCACTGTTACTAGC

Supplementary Table 2. Comparison of senescence markers in T2DM participants on GLP1R-agonists versus those not on these drugs. mRNA data are normalized values ($x10^{-4}$).

	GLP1R-users (N=12)	GLP1R-non users (N=15)	P-value
p16_variant 1+5	6.06 (4.22-9.33)	4.78 (3.55-4.94)	0.205
p16_variant 5	6.38 (3.29-9.25)	6.23 (5.14-7.37)	0.807
$p21^{Cip1}$	2.45 (2.00-2.51)	1.95 (1.68-2.30)	0.157
CD28	2.86 (1.58-3.58)	2.81 (1.90-3.21)	0.845
**HbA1c**	7.60 (7.18-7.93)	7.60 (7.20-8.15)	0.607
**Skin Ages**	2.80 (2.65-3.10)	2.90 (2.50-3.40)	0.806

Supplementary Table 3. Skeletal parameters in the T2DM patients stratified by tertiles of expression of T-cell *p16_variant 1+5* mRNA levels. P-value is from the Kruskal-Wallis test evaluating whether there is a at least one group that is different from the others.

	T1 (N = 9)	T2 (N = 9)	T3 (N = 9)	P-value
**DXA BMD**				
Femur Neck (g/cm^2)	0.954 (0.947, 1.076)	0.968 (0.898, 1.121)	0.891 (0.873, 1.062)	0.447
Total Femur (g/cm^2)	1.11 (1.01, 1.18)	1.05 (0.97, 1.18)	1.03 (0.96, 1.19)	0.783
Spine-L1-L4 (g/cm^2)	1.39 (1.34, 1.48)	1.23 (1.18, 1.71)	1.14 (1.11, 1.22)	0.147
Spine-L1-L4 TBS	1.36 (1.29, 1.38)	1.43 (1.32, 1.46)	1.35 (1.28, 1.38)	0.612
Forearm Radius UD (g/cm^2)	0.491 (0.432, 0.497)	0.445 (0.378, 0.503)	0.453 (0.444, 0.554)	0.418
Forearm Total Radius (g/cm^2)	0.723 (0.662, 0.728)	0.646 (0.607, 0.675)	0.690 (0.662, 0.728)	0.082
**HRpQCT Ultradistal Radius**				
Trabecular BV/TV	0.232 (0.215, 0.243)	0.236 (0.215, 0.276)	0.225 (0.199, 0.258)	0.841
Trabecular Number (1/mm)	1.58 (1.54, 1.70)	1.55 (1.42, 1.75)	1.59 (1.49, 1.69)	0.955
Trabecular Thickness (mm)	0.227 (0.223, 0.232)	0.243 (0.236, 0.248)	0.231 (0.227, 0.241)	0.140
Trabecular Separation (mm)	0.601 (0.571, 0.623)	0.609 (0.552, 0.698)	0.601 (0.562, 0.671)	0.977
Stiffness (kN/mm]	65.8 (50.5, 68.8)	58.2 (49.7, 64.6)	56.1 (47.8, 66.4)	0.665
Failure load (kN)	3.53 (2.83, 3.74)	3.18 (2.74, 3.54)	3.10 (2.50, 3.68)	0.801
**HRpQCT Distal Radius**				
Cortical vBMD (mg HA/cm^3)	1026 (970, 1053)	980 (967, 1007)	1014 (1010, 1031)	0.635
Cortical Area (mm^2)	77.8 (74.9, 81.4)	69.9 (66.8, 78.3)	73.7 (69.3, 78.1)	0.206
Cortical thickness (mm)	2.13 (2.03, 2.31)	1.97 (1.84, 2.02)	2.00 (1.84, 2.07)	0.095
Periosteal Perimeter (mm)	47.2 (46.0, 50.3)	47.9 (46.4, 48.7)	49.3 (46.2, 52.1)	0.825
Intra-Cortical Porosity	0.019 (0.008, 0.023)	0.010 (0.009, 0.014)	0.008 (0.007, 0.009)	0.305
**HRpQCT Ultradistal Tibia**				
Trabecular BV/TV	0.261 (0.216, 0.286)	0.292 (0.275, 0.305)	0.260 (0.240, 0.292)	0.198
Trabecular Number (mm^{-1})	1.44 (1.39, 1.76)	1.66 (1.57, 1.69)	1.59 (1.38, 1.69)	0.682
**Trabecular Thickness (mm)**	**0.252 (0.245, 0.280)**	**0.282 (0.278, 0.296)**	**0.259 (0.248, 0.277)**	**0.024**
Trabecular Separation (mm)	0.698 (0.571, 0.729)	0.608 (0.595, 0.640)	0.615 (0.590, 0.709)	0.654
Stiffness (kN/mm]	176.7 (170.6, 182.0)	179.2 (166.6, 192.5)	157.8 (154.8, 184.9)	0.618
Failure load (kN)	9.72 (9.47, 9.86)	9.65 (9.1, 10.4)	8.72 (8.37, 10.12)	0.669
**HRpQCT Distal Tibia**				
Cortical vBMD (mg HA/cm^3)	956 (918, 969)	900 (886, 936)	937 (880, 984)	0.377
**Cortical Area (mm^2)**	**180 (166, 181)**	**174 (171, 180)**	**159 (156, 163)**	**0.009**
**Cortical thickness (mm)**	**2.52 (2.35, 2.68)**	**2.61 (2.48, 2.76)**	**2.30 (2.18, 2.36)**	**0.025**
Periosteal Perimeter (mm)	87.1 (83.7, 88.0)	86.3 (83.6, 87.7)	84.8 (81.7, 87.2)	0.796
Intra-Cortical Porosity	0.029 (0.022, 0.033)	0.032 (0.025, 0.044)	0.027 (0.022, 0.031)	0.191
